# Evaluation of hop test movement quality to enhance return to sport testing. A cross-sectional study

**DOI:** 10.3389/fspor.2024.1305817

**Published:** 2024-03-04

**Authors:** Melanie Weber, Mirjam Müller, Moritz Mathieu-Kälin, Sandro Caminada, Marina Häberli, Heiner Baur

**Affiliations:** ^1^ Division of Physiotherapy, Department of Health Professions, Bern University of Applied Sciences, Bern, Switzerland; ^2^ Altius Swiss Sportmed Center AG, Rheinfelden, Switzerland

**Keywords:** movement quality, hop test, anterior cruciate ligament, return to sport, reliability

## Abstract

**Introduction:**

Return to Sport tests with functional hop tests are often used to decide when a person is ready to return to sport after an anterior cruciate ligament (ACL) injury. Poor movement quality, such as knee valgus, hip adduction and hip internal rotation is considered a risk factor for ACL injury. However, it is unclear whether existing tests adequately cover the aspect of movement quality. This study aims to investigate whether there is a relationship between the calculated limb symmetry index (LSI) of hop tests as an indication of performance and the total score of the “Quality First” assessment (movement quality). The second aim is to examine the reliability of the newly developed “Quality First” assessment for evaluating movement quality in hop tests.

**Methods:**

The cross-sectional study recruited 34 patients with an ACL reconstruction. The vertical hop, single-leg hop for distance, and side hop tests were performed and recorded. The video recordings were assessed using the “Quality First” assessment. The Spearman correlation coefficient was calculated using the LSI and the “Quality First” total score. Intraclass correlation coefficients (ICC) and standard error of measurements (SEM) were used to calculate intra- and interrater reliability. In addition, the minimal detectable change (MDC) was determined.

**Results:**

The correlation test between the LSI and the “Quality First” total score showed no correlation for all three jumps (*r* = −0.1–0.02/*p*-value = 0.65–0.93). The interrater reliability of the “Quality First” assessment showed fair to good reliability (ICC_2_: 0.45–0.60), with SEM ranging from 1.46 to 1.73 and the MDC from 4.06 to 4.8. Intrarater reliability was good to excellent (ICC_3_: 0.73–0.85), with SEM values ranging from 0.89 to 1.09 and the MDC from 2.47 to 3.01.

**Conclusion:**

The quality of movement, measured with the “Quality First” assessment, indicated no correlation with the calculated LSI from jump performance, therefore movement quality should also be examined in Return to Sport tests. The “Quality First” assessment shows fair to good reliability when used by different raters. When used multiple times by the same rater, the assessment has good to excellent reliability.

## 1 Introduction

Injuries to the anterior cruciate ligament (ACL) are common in athletes. In 2020, 7,330 people were hospitalized for a reconstruction of the ACL in Switzerland, which has 8.6 million residents (1). Most ACL injuries occur during deceleration, lateral pivoting, or landing tasks and are often non-contact injuries (2). ACL reconstructions (ACLr) has become a standard treatment for people who want to return to high-risk sports, including movements such as jump landing, pivoting, and cutting (3–5).

A significant difficulty during ACL rehabilitation is deciding when it is safe for a patient to return to sport (3, 6, 7). Return to sport is defined as “the athlete has returned to his or her defined sport, but is not performing at his or her desired performance level” (8) (p. 854). Therefore a Return to Sport test (RTS) battery is often conducted during the final stage of rehabilitation, typically between five to ten months after surgery (9). The purpose of RTS tests is to decide the point at which re-entry into the sport can be permitted with a reduced risk for a second ACL injury (9). In addition to strength tests and self-reported knee function, RTS test batteries often include single-leg hop tests to evaluate functional performance (4, 10, 11). As athletes move in multiple directions during pivoting or cutting sports, it is recommended to test different movement directions (12). The combination of the vertical hop (VH), single-leg hop for distance (SLHD), and side hop (SH) test covers the different directions of movement in sports and has a high ability to discriminate between the injured and uninjured leg (5, 10). These should therefore be included in the RTS evaluation (5, 10).

After these functional hop tests, the limb symmetry index (LSI) is commonly calculated to decide if a person should return to sport. For comparison, the uninjured limb is used as a control (6, 11). The LSI is calculated by dividing the hop performance of the injured leg by the uninjured leg and multiplying by 100 to obtain a percentage (10, 11). If a side-by-side difference is greater than or equal to 90%, the LSI is classified as satisfactory, and a return to sport is recommended (4, 10, 11, 13).

However, the rate of a second ACL rupture is reported to be 20%–40% despite performing these functional RTS tests and reaching the predefined LSI values (4, 14–16). These high rates of injury reoccurrence indicate a significant risk for second knee injury for all athletes who return to pivoting or cutting sports after an ACLr (17). One possible explanation is that the uninjured leg may be an inappropriate control for the test (6). Hiemstra et al. demonstrated that the contralateral limb also displays deficits, such as reduced strength after an ACL injury (18). Gokeler et al. observed that the unaffected leg demonstrated reduced jump distance, in comparison to the metrics of a healthy control group (6). Therefore, the evaluation of the LSI seems to overestimate the function of the injured limb and produces a misleading test result (19, 20). Furthermore, the calculated LSI does not guarantee that the injured limb has returned to the same, preoperative level because both limbs are deficient and display weaker movement control in the hop test performance (6, 19, 20). This suggests that the current RTS evaluation, which calculates the LSI of different single-leg hop tests, is insufficient to determine whether an athlete is safe to return to sport (20).

Moreover, poor dynamic movement control during a jump landing is considered a risk factor for an ACL injury (21, 22). Increased dynamic knee valgus, decreased knee and hip flexion angle with simultaneous hip joint internal rotation, and hip adduction during jump landings are movements that pose a risk for an ACL injury (2, 22–24). Goerger et al. demonstrated that the biomechanics of the lower limbs are altered after an ACLr. They found an increased knee valgus and hip adduction at the initial contact of a jump landing compared to a control group. Additionally, during the landing, there was a significant decrease in the peak internal knee extension moment and peak internal hip flexion moment (25). However, the LSI of hop tests does not take movement quality into account (26). Furthermore, the symmetry of jump performance in hop tests does not provide information on knee function recovery and movement control during jump landing, which are associated with increased risk of ACL injury (26). Therefore, the evaluation of movement quality during jump landing seems to be relevant for the prevention of a second ACL injury (5). Several studies recommend including the evaluation of movement quality in hop tests in the RTS decision to reduce the high risk of a second ACL injury (11, 14, 26, 27). Currently, no instrument exists to evaluate movement quality in SLHD, VH, and SH tests without the use of advanced techniques such as three-dimensional motion analyses (28).

Therefore, the aim of this study is to determine whether there is a correlation between the LSI percentage value as an indicator for performance and the total score of the “Quality First” assessment as an indicator for movement quality (29) Furthermore the question if patients who achieve an LSI ≥ 90% have higher “Quality First” total scores than those with an LSI lower than 90% is investigated. This study hypothesizes that there is no correlation between the LSI and the estimated movement quality. This hypothesis based on the findings of a systematic review that there is no correlation between the limb symmetry in hop distance and the biomechanical knee function after ACLr (26). Despite good limb symmetry in hop distance, an athlete might have poor knee biomechanics for the injured limb during landing (19, 26, 30). This result would reinforce the need to assess the movement quality of the lower limb during jump landings separately and promotes the integration of this evaluation into RTS testing to prevent second ACL injuries.

The second aim of this study is to investigate the inter- and intrarater reliability of the “Quality First” assessment, which was recently developed for this study (29).

## 2 Methods

### 2.1 Participants

For this cross-sectional study, 34 participants who underwent ACLr (18 males, 16 females; age, 24.2 ± 8.2 years; height, 173.4 ± 8.5 cm; weight, 71.2 ± 12.1 kg) were recruited between February 2021 and March 2022 from the Altius Swiss Sportmed Center AG in Rheinfelden, Switzerland. The responsible ethical committee (Ethikkommission Nordwest- und Zentral-Schweiz, EKNZ) approved the study with the reuse of routine data (EKNZ no.: 2021-01169).

Patients were included in the study if they had an ACLr at least six months prior to data collection. Participants completed a minimum of one RTS test during rehabilitation at the Altius Swiss Sportmed Center AG (Rheinfelden, Switzerland), and they declared voluntary participation (by signing an informed general consent form). Participants’ ACLr was on average 9.4 ± 2.9 months before the RTS test. The SH test was conducted for the first time during the nine months assessment. Individuals undergoing testing earlier than nine months postoperatively were therefore exempted from the SH test due to safety reasons and had only de VH and SLHD test. Some patients had additional injuries, mostly treated surgically alongside the ACLr. In total, 19 patients suffered from additional injuries such as lateral meniscus tear (*n* = 10), medial meniscus tear (*n* = 11), lateral collateral ligament injury (*n* = 1), medial collateral ligament injury (*n *= 3), cartilage damage (*n* = 2), bone bruise (*n* = 1), and other injuries (*n* = 3). Twenty-nine patients had an ACLr using a semitendinosus graft, and five patients had an ACLr using a quadriceps tendon graft. The mean Tegner Score was 7.6 ± 1.1. The characteristics of the participants are presented in Supplementary Table S1. Participants with the inability to perform a single-leg hop were excluded from the study.

### 2.2 Procedure

Data collection took place at the Altius Swiss Sportmed Center AG in Rheinfelden, Switzerland. All participants were informed about the study procedure. Before the hop tests, all participants completed a warm-up session, which consisted of 5–10 min of cycling. All tests were conducted by two trained movement scientists who were not involved in the subsequent descriptive video analysis.

The testing included three single-leg hop tests: the vertical hop, single-leg hop for distance, and side hop test, according to Gustavsson et al. [10]. These tests are all part of the routine RTS protocol at the Altius Swiss Sportmed Center AG. All participants wore their own sports shoes during testing. For each hop test, participants could perform as many practice jumps as needed. After that, a maximum of three test trials for each leg were conducted for the VH and SLHD tests.

During the VH test, participants stood on the test leg, jumped as high as possible, and landed on the same leg. For the SLHD test, participants stood on the test leg, jumped as far as possible, and landed with the same leg. For both these hop tests, participants' hands were unconstrained, and the landing position needed to be held stable for at least 2 s. The hop was considered unsuccessful if the contralateral limb touched the floor within the first 2 s after landing. For the SH test, participants stood on the test leg and jumped from side to side over two parallel strips, placed 40 cm apart. Participants were required to jump as many times as possible from side to side for 30 s, with their hands placed on their hips. An attempt was considered invalid as soon as the foot touched the marking line. For each hop test, the uninjured leg was tested first.

#### 2.2.1 Development of the “Quality First” assessment

Three experienced physical therapists conducted an extensive literature review to determine the biomechanical risk factors for a non-contact ACL injury. This literature was used to develop an instrument that measures the movement quality of hop tests. Seven external physiotherapists completed test evaluations on the first draft of the “Quality First” assessment, and their feedback was used to create a more feasible and user-friendly assessment. After a concluding consensus meeting, the final version of the “Quality First” assessment was agreed (Supplementary Figure S1) (29). The assessment was created for the three subgroups: VH, the SLHD, and the SH. Through this battery of hop tests, movements in different dimensions are recorded (similar to movements in sports) (27). In addition, the SH test assesses jumping behavior under possible muscle fatigue (5). A two-dimensional video recording was sufficient for evaluating the “Quality First” assessment. That is the prerequisite for a low-cost, feasible, and easy to implement tool resulting in a potentially high clinical relevance for rehabilitation practice.

### 2.3 Data collection

#### 2.3.1 Evaluation of movement quality

Frontal plane video analysis was used to measure the movement quality in the hop tests. The videos were recorded with an iPad® Pro 11.0 (Apple Inc., Cupertino, USA) and the installed Dartfish®-Application (Dartfish® 360, Dartfish HQ, Lausanne, CH). The movement quality was evaluated with the “Quality First” assessment (29). All videos were evaluated by three experienced physiotherapists with 3–4 years of professional experience especially in sports physiotherapy. The kinematics (shock absorption, trunk, hip, knee, and ankle position) were assessed at initial contact until the body's lowest center of gravity point was reached. It was important to evaluate this movement phase because it is when most non-contact ACL injuries occur (31). The “Quality First” SLHD and VH tests incorporate the evaluation of eight different items, while the SH test uses six items. Detailed descriptions of these items are provided in Supplementary Figure S2. Each item can be awarded between zero and three points. A higher score indicating good movement quality patterns, and zero points indicating a poor landing technique. The “Quality First” total score is the sum of all the items. A patient can achieve a maximum score of 24 points for the VH and SLHD tests and 18 points for the SH test.

#### 2.3.2 Evaluation of the hop test performance

The maximum jump height was assessed to measure the performance of the VH test. An inertial measurement unit sensor (Axiamo GmbH® X22 Motion Sensing Technology, 2020, Nidau, Switzerland) was used for this evaluation. This sensor was placed on the tip of the participant's shoes during the VH tests. For the SLHD test, the participant performed three successful trials on each leg, and the jump distance was evaluated for each hop. The distance between the toe and heel was then measured. For each leg, the jumps used in the analysis for the VH and SLHD tests were the highest and farthest jumps, respectively. The SH test was completed once for each leg, and the number of valid jumps completed within 30 s was noted. The number of valid jumps with the left and the right leg was used for the evaluation.

### 2.4 Data analysis

#### 2.4.1 Comparison of LSI and “Quality First” total score

The maximum jump height (for the VH test), the maximum jump distance (for the SLHD test), and the maximal number of side hops in 30 s (for the SH test) were used to calculate the LSI percentage. For this percentage, the jump height, jump distance or number of side hops from the injured leg was divided by the values of the uninjured leg and multiplied by 100 (11). The sum of the “Quality First” assessment for the injured leg was used to analyze the correlation between the LSI percentage and the movement quality as assessed by “Quality First”.

#### 2.4.2 Inter- and intrarater reliability of the “Quality First” assessment

Each hop test video was analyzed with the “Quality First” assessment. The rater was unaware of which leg was injured. During the evaluation of the jump recordings, raters were allowed to pause, rewind, or watch the jump in slow-motion. Raters evaluated the trunk, hip, knee, and foot position during landing and points between zero to three were awarded. The total score of the “Quality First” assessment was used for the statistical analysis.

To assess the intrarater reliability of the “Quality First” assessment, rater 1 analyzed the videos twice, with a wash-out phase of a minimum of two weeks between the evaluations (32). The interrater reliability of “Quality First” was assessed by three raters, who were each unaware of the scores of the other raters. Before this evaluation, the scoring of movement possibilities for landings was defined in a consensus meeting, where all the answer options for each item and hop test were discussed.

### 2.5 Statistical analyses

All statistical analyses were carried out using the RStudio software for Windows (Version 3.6.1, License AGPL, Boston, USA). The analyses of the movement quality and jump performance was performed for each of the three hop tests (SLHD, VH, SD). Descriptive statistics, including the mean, standard deviation (SD), and 95% confidence interval (CI) were used to present the participants' characteristics. The normal distribution of data was controlled by a Q-Q plot.

A Spearman correlation analysis was used to determine the correlation between the LSI and the “Quality First” result from the injured leg. A correlation coefficient value of 0.00–0.2 is negligible, 0.21–0.40 is weak, 0.41–0.60 is moderate, 0.61– 0.80 is strong, and 0.81–1.00 is a very strong correlation (33). In addition, separate boxplots were used to present the distribution of “Quality First” total scores for the group with an LSI ≥ 90% and for the group with an LSI < 90%. A Welch-test (for unequal variances) and a *t*-test (for equal variances) was calculated to examine if the mean “Quality First” total scores differed between the two groups. A *p*-value of ≤0.05 was used to indicate statistical significance for all analyses.

The intrarater reliability of the “Quality First” assessment was investigated by calculating the two-way mixed-effects model intraclass correlation coefficient (ICC_3_) with the 95% confidence interval. For the interrater reliability, the two-way random-effects model intraclass correlation coefficient (ICC_2_) was used. Thus the reliability results can be generalized to any rater (34). The standard error of measure (SEM) was calculated using the following equation (35): $\mathrm{SEM}=\mathrm{SD}*\;\sqrt{1-\mathrm{ICC}}$. The minimal detectable change (MDC) was calculated with the formula 1.96∗SEM∗√2 in RStudio (36). An ICC value of 0.00–0.40 indicates poor reliability, 0.40–0.75 indicates fair to good reliability, and 0.75–1.00 indicates excellent reliability (37).

## 3 Results

Thirty-four patients met the eligibility criteria and were included in the study. From these patients not all of them completed all three types of hop tests. A sample of 25 patients completed the VH test, 25 patients had done SLHD test, and a total of 25 patients completed the SH test (all tests completed with the right and left leg). Therefore, fifty jumps for each hop test could be analyzed. According to the COSMIN guidelines, this is an adequate number of values for assessing reliability (38) The normal distribution of the data was approved with a Q-Q plot. All data displayed a normal distribution which was presented with an almost straight line in the plot.

### 3.1 Descriptive statistics

The minimum and maximum score and the mean value of the “Quality First” total scores were calculated for all three raters. The comparison of the mean values of the raters showed that rater 1 had slightly lower mean values than rater 2 and rater 3. The mean values of rater 1 ranged from 9.0–15.4, rater 2 from 11.7–16.6 and rater 3 showed mean values from 11.1–16.3 (Table 1). The mean values of the “Quality First” total score as well as minimum and maximum score were calculated for the descriptive statistics of the intrarater reliability. The mean values of the first evaluation ranged from 14.5–20.1 and were similar to the second evaluation, where the values ranged from 14.7–20.1 (Table 2).

**Table 1:** Descriptive statistics “Quality First” total scores (interrater reliability).

Total “Quality First” scores for each rater for each hop test									
						95% CI			
		*N*	Mean	SD	SE	Lower bound	Upper bound	Min	Max
rater1 (3. Evaluation)	VH	50	15.4	2.1	0.3	14.58	16.03	11	21
	SLHD	50	15.0	2.1	0.3	14.38	15.54	12	20
	SH	50	9.0	2.4	0.3	8.33	9.63	4	14
rater2	VH	50	16.6	3.0	0.4	15.75	17.41	8	22
	SLHD	50	16.1	2.6	0.4	15.37	16.79	10	22
	SH	50	11.7	2.5	0.4	11.04	12.44	6	16
rater3	VH	50	16.3	3.2	0.5	15.37	17.15	11	24
	SLHD	50	14.9	2.8	0.3	14.24	15.70	9	21
	SH	50	11.1	2.2	0.3	10.50	11.74	6	17

VH, vertical hop; SLHD, single-leg hop for distance; SH, side hop; *N*, number; Std. Deviation, standard deviation; Std. Error, standard error; CI, confidence interval; Min, minimum; Max, maximum.

**Table 2:** Descriptive statistics “Quality First” total scores (intrarater reliability).

“Quality First” total scores (intrarater reliability)									
						95% CI			
		*N*	Mean	SD	SE	Lower bound	Upper bound	Min	Max
Rater 1 (1. Evaluation)	VH	50	19.7	2.4	0.3	18.99	20.33	13	23
	SLHD	50	20.1	2.0	0.3	19.49	20.63	15	24
	SH	50	14.5	1.7	0.2	14.01	14.95	10	17
Rater 1 (2. Evaluation)	VH	50	19.8	2.7	0.4	19.09	10.59	14	24
	SLHD	50	20.1	2.2	0.3	19.50	20.70	15	24
	SH	50	14.7	2.0	0.3	14.20	15.28	10	18

VH, vertical hop; SLHD, single-leg hop for distance; SH, side hop; *N*, number; SD, standard deviation; SE, standard error; CI, confidence interval; Min, minimum; Max, maximum.

### 3.2 Correlation of LSI and “Quality First” total scores

#### 3.2.1 Vertical hop

Twelve patients (48%) of the twenty-five had a hop symmetry of ≥90% for the VH test. The mean LSI values were 98.8 ± 5.9 and 76.1 ± 10.7 for the group with an LSI ≥ 90% and LSI < 90%, respectively (Supplementary Table S3).

**Figure 1:**
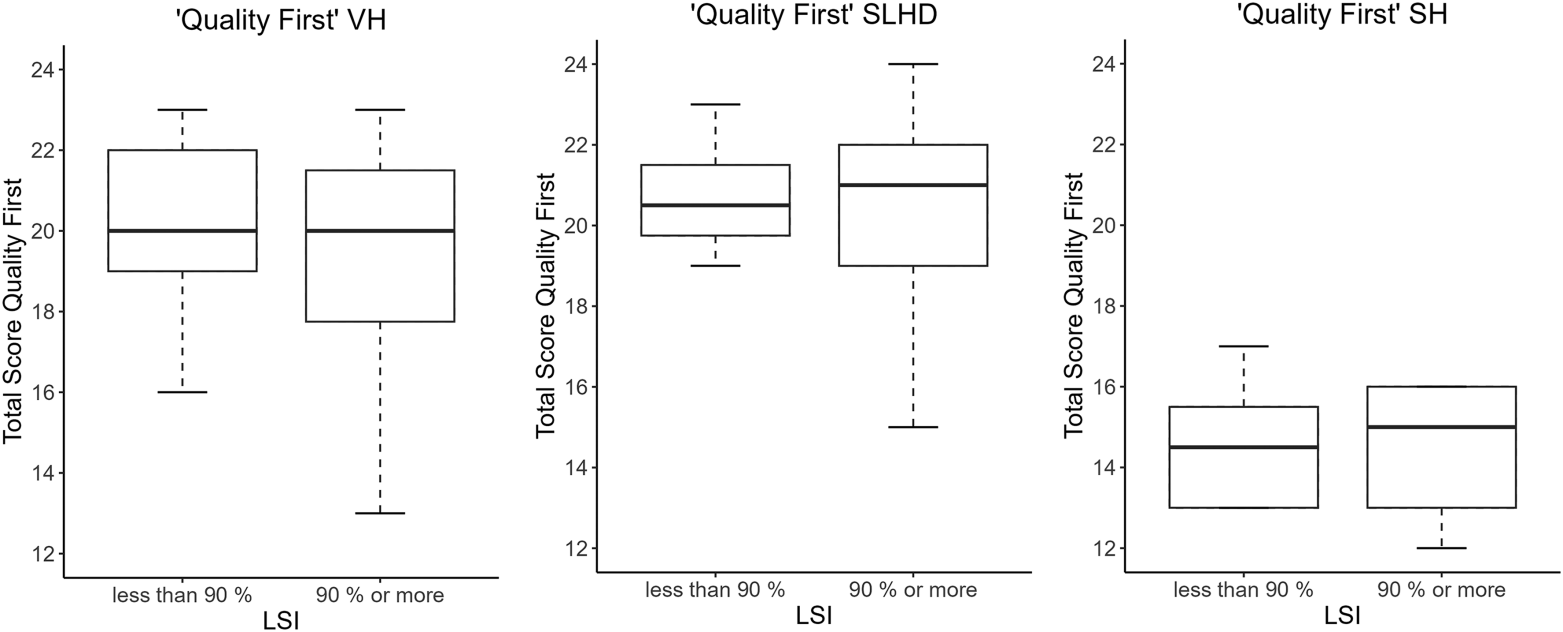
Distribution “Quality First” total scores in two differnt grops.

The Spearman correlation coefficient revealed no statistically significant correlation between the “Quality First” total score of the operated leg and the calculated LSI (Table 3). The mean difference of the “Quality First” VH test total score of the patients who achieved an LSI ≥ 90% (19.3 ± 3.2) and those who had an LSI < 90% (20 ± 2.1) was calculated with the Welch-Test. This test showed no statistically significant difference between the mean scores *p*-value of 0.55 (Supplementary Table S4). The distribution of the “Quality First” total scores of the two groups is illustrated in the boxplot in Figure 1.

**Table 3:** Correlation LSI and “Quality First” total score.

Spearman correlation coefficient: LSI and “Quality First"		
	rho	*p*-value
Vertical hop	0.02	0.93
Single-leg hop for distance	−0.10	0.65
Side hop	0.04	0.87

LSI, limb symmetry index.

#### 3.2.2 Single-leg hop test for distance

For the SLHD, 21 participants (84%) passed the cut-off value of LSI ≥ 90%. The mean LSI value was 99.6 ± 5.0 and 85.8 ± 5.2 for the group LSI ≥ 90% and the group LSI < 90%, respectively (Supplementary Table S3).

The Spearman correlation coefficient of the “Quality First” total scores of the injured leg and the calculated LSI were not statistically significant (Table 3). The group with an LSI ≥ 90% had a mean “Quality First” total score of 20 ± 2.43, and the group with an LSI < 90% had a mean “Quality First” total score of 20.75 ± 1.71.

The difference in the mean “Quality First” total scores of both groups was calculated with the Welch-Test. This result showed no statistically significant difference between the mean with a *p*-value of 0.49 (Supplementary Table S4). The distribution of the “Quality First” total scores of the two groups is illustrated in the boxplot in Figure 1.

#### 3.2.3 Side hop

For the SH test, 13 participants (52%) reached the cut-off value of LSI ≥ 90%. The mean LSI value in the group with an LSI ≥ 90 was 102.6 ± 9.6. The mean LSI value in the group with an LSI < 90% was 76.8 ± 13.1 (Supplementary Table S3).

The Spearman correlation coefficient was calculated with the “Quality First” total scores of the injured leg and the LSI values of the SH test. There was no statistically significant correlation (Table 3) The mean “Quality First” total score was 14.5 ± 1.6 in the group with an LSI ≥ 90% and 14.6 ± 1.7 in the group with an LSI < 90%.

The difference between the mean “Quality First” total score of the two groups was calculated with an independent t-test. This result showed no statistically significant difference between the mean scores with a *p*-value of 0.85 (Supplementary Table S4). The distribution of the “Quality First” total scores of the two groups are illustrated in the boxplot in Figure 1. All illustrations of the correlations are presented in the Supplementary Figure S2.

### 3.3 Inter- and intrarater reliability

#### 3.3.1 Interrater reliability

The overall mean value of the “Quality First” total scores of the VH, SLHD, and SH tests for the interrater reliability was 16.1 ± 2.8, 15.3 ± 2.6, and 10.6 ± 2.6, respectively (Supplementary Table S2). The ICC values of the interrater reliability ranged from 0.45–0.60 and indicated fair to good reliability. The SEM values ranged from 1.46–1.73, and the MDC values ranged from 4.06–4.80. The values of the different “Quality First” assessments with the 95% CI are presented in Table 4.

**Table 4:** Interrater reliability.

Interrater reliability after consensus meeting				
	ICC_2_	(95% CI)	SEM	MDC
“Quality First” vertical hop	0.60	(0.45–0.74)	1.73	4.79
“Qualiry First” single-leg hop for distance	0.50	(0.33–0.65)	1.73	4.80
“Quality First” side hop	0.45	(0.14–0.67)	1.46	4.06

ICC_2_, intraclass correlation coefficient; CI, confidence interval; SEM, standard error of measurement; MDC, minimal detectable chang.

#### 3.3.2 Intrarater reliability

The overall mean value of the “Quality First” total score of the VH, SLHD, and SH tests for the intrarater reliability were 19.8 ± 2.6, 20.1 ± 2.1, and 14.6 ± 1.8, respectively (Supplementary Table S2). The ICC values for the intrarater reliability of the “Quality First” assessments ranged from 0.73–0.85 and indicated good to excellent reliability. The SEM values ranged from 0.89–1.09, and the MDC values ranged from 2.47–3.01. The values of the different “Quality First” assessments with the 95% CI are presented in Table 5.

**Table 5:** Intrarater reliability.

Intrarater reliability				
	ICC_3_	95% CI	SEM	MDC
“Quality First” vertical hop	0.85	(0.75–0.91)	0.98	2.73
“Quality First” single-leg hop for distance	0.73	(0.57–0.84)	1.09	3.01
“Quality First” side hop	0.76	(0.62–0.86)	0.89	2.47

ICC_3_, intraclass correlation coefficient; CI, confidence interval; SEM, standard error of measurement; MDC, minimal detectable change.

## 4 Discussion

The purpose of this study was to analyze if the calculated LSI of the VH, SLHD, and SH tests correlate with the movement quality evaluation. The second aim was to examine the inter- and intrarater reliability of the newly developed “Quality First” assessment. First, this study found a poor correlation between the calculated LSI and the movement quality evaluation from the VH, SLHD, and SH tests. This study demonstrated also that the “Quality First” assessment displays good to excellent intrarater reliability and fair to good interrater reliability for evaluating patients after an ACLr.

The hypothesis that the calculated LSI does not correlate with the movement quality of a hop test landing was confirmed with the present study. The Spearman correlation coefficient calculation demonstrated a negligible correlation in all subgroups of the “Quality First” assessment. It indicates no association between the “Quality First” total scores and the limb symmetry in jump performance. There was not a higher mean total score for the “Quality First” assessment in the group with an LSI ≥ 90% than in the group with an LSI < 90%. The findings of this study indicated that the movement quality is not related to the hop performance. In other words, if a hop performance is judged as successful with reaching an LSI ≥ 90%, it does not automatically predict satisfactory landing kinematics. Several studies confirm the findings of the present study. Gokeler et al. found that many patients achieve normal hop distance with the injured leg in the SLHD test after an ACLr, but the movement kinematic still showed deficits (15). Other studies concluded that many people had abnormal landing kinematics on hop tests after an ACLr compared to the uninjured leg, and still achieved an LSI of 90% or more (11, 39). Several studies found that the symmetry of the hop distance does not indicate ideal kinematics or readiness for a return to sport (19, 30). One study investigated whether achieving an LSI ≥ 90% from hop tests is associated with secondary ACL injuries. They found that the LSI of hop tests alone did not distinguish between patients who did and did not have a second ACL injury (16). These findings might indicate that the evaluation of the movement quality assesses different aspects than the evaluation of the leg symmetry. This finding might explain the high rate of second ACL injuries (26). As mentioned in the introduction, risk factors for ACL injuries, are poor movement kinematics during landing tasks (22). According to the results of this study, the quality of movement is not captured by the calculated LSI. It should therefore be assessed as supplementary. It suggests that the movement quality of a hop landing should be evaluated beside the calculated LSI of jump performance during RTS tests (15, 40).

Padua et al. investigated the interrater reliability of a similar assessment (41). The landing error scoring system (LESS) investigates different body parts during bipedal landing tasks. The study of Padua et al. showed an ICC value of 0.84 and an SEM value of 0.71 for the interrater reliability, which indicated better reliability than the present study. In contrast to the “Quality First” assessment, the items of the LESS have just two answer options: “yes” and “no”. The “Quality First” assessment tool has four scoring options such as “knee joint is neutrally aligned”, “slight knee joint valgus”, “clearly knee joint valgus”, “extreme knee joint valgus”. Reducing the number of possible answers per item might increase the agreement and thus the ICC value. This hypothesis is supported by several studies, which concluded that dichotomous rating scales increased reliability (42–44). The participants in the study of Padua et al. were healthy subjects with no orthopedic injuries. In contrast, the present study examined patients after an ACLr. Although the injured and uninjured leg was tested with the “Quality First” assessment, both sides may show changes in movement quality after an ACLr (6). Thus, comparing the results of these studies is difficult.

Weir et al. showed worse interrater reliability values than the present study, even though this study also used a 4-point scale with a similar number of participants (*n* = 40) (45). Weir et al. conducted a video analysis of different dynamic core stability tests in real-time. The interrater ICC values of the various core stability tests ranged from 0.09–0.51, which were interpreted as poor (45). One difference was found between these studies: Weir et al. evaluated real-time video analysis, while the present study used the slow-motion function (45). Some assumptions are that the movement quality evaluation of static tests had better reliability than dynamic tests (45). Therefore, using the slow-motion and pause function to analyze hop landings might enhance raters' agreement because converts the dynamic into a static position and allows multiple views (42). In contrast to this expectation, the evaluation of the hop tests was subjectively not easier with the slow-motion function. There were difficulties in deciding which precise millisecond of the landing movement should be evaluated. This aspect might provoke variances between the scoring of the raters.

Working with the “Quality First” assessment has challenging aspects. The evaluation of movement quality with video analysis incorporates always some degree of subjectivity in the rating (46). The interpretation of whether there was a normal landing movement or a risky movement remains subjective. There were efforts to reduce the subjectivity of the “Quality First” assessment. Some of the assessment criteria were more precisely described than others. For example, the item knee valgus had a more precise description than the hip rotation item. It has to be acknowledged, that subjective interpretation will always influence the evaluation (47). Imprecisely described items may underlie reduced interrater reliability (42). There were more possibilities for differences in the evaluation between the raters mentioned in the literature. For example, the raters' experience with evaluating hop tests and rater training could influence variations in raters' agreement (43). Therefore, a training session or a manual with detailed written information about the “Quality First” scoring might improve agreement among the raters (see item description in the “Quality First” assessment in the Supplementary Material).

Overall, the intrarater reliability of the “Quality First” assessments were higher than the interrater reliability values. Several studies had similar findings (41, 45, 48). The intrarater reliability for the “Quality First” subgroups, vertical hop, and side hop demonstrated greater ICC values than 0.75 and indicated excellent intrarater reliability. The ICC value of the “Quality First” subgroup single-leg hop for distance test had a slightly lower ICC value of 0.73, which nevertheless showed a good intrarater reliability. These findings suggest that the “Quality First” assessment with the VH, SLHD, and SH tests were reliable when used by the same rater for repeated hop evaluations. Furthermore, the ICC values of Padua et al. with the LESS screening tool, showed slightly higher ICC values (0.91) than the present study (41). This difference may be due to the number of response options described above. One study found similar ICC values to this study. Three experienced scorers investigated the intrarater reliability of the Balance Error Scoring System (BESS) by evaluating 30 videos of people performing the BESS balance positions (48). This study showed an ICC value of 0.74 of the BESS total score, which is comparable to the intrarater ICC values of the current study (48). This study also had more than two response possibilities, similar the present study.

The MDC for the “Quality First” assessment was calculated. The MDC indicates the minimum number of points required to represent a real change in landing kinematics (36). The interrater “Quality First” total score calculation of the MDC across the three raters after the consensus meeting ranges from 4.06 to 4.8 points. Therefore, the total score of the “Quality First” assessment should increase from 4.06 to 4.8 points from the baseline evaluation and the subsequent hop evaluation. This point difference indicated a real change in landing kinematics and is not attributed to variability in scoring. The “Quality First” intrarater MDC ranges from 2.47 to 3.01 points. Therefore, the increase in the “Quality First” total score should be 2.47 in the VH test and 3.01 in the SLHD test on a 24-point scale, to detect a real change. A change of 2.47 points in an 18-point scale is necessary to detect a real change in movement quality for the SH test. One study had similar findings to the present study. They showed a higher value for the mean interrater MDC (9.4 points of a maximum possible score of 60 points) than the mean intrarater MDC (7.3 points) (48). They justified this MDC difference with a greater interrater scoring variability, which is also a plausible explanation for the present study. These calculations of the MDC values were necessary for the clinical use of the “Quality First” assessment.

The present study had some strengths and limitations. A strength of this study is that the evaluation of the movement quality of hop landings with the “Quality First” assessment is user friendly and straightforward and can be conducted in every clinical practice. Furthermore, the participants were heterogeneous, with an almost equal number of men (*N* = 18) and women (*N* = 16) and an age range from 13 to 43 years (see Supplementary Figure S1). In addition, the “Quality First” assessment was used for the injured and uninjured leg. Thus, the data are transferable to a broad population.

One limitation is that the video recordings were only from a frontal view. That made evaluating of the items hip flexion, and knee joint flexion challenging and could cause differences in the ratings of these items by the three raters. Just due to the fact that the single frontal view is not adequate for proper evaluation of these movement aspects. Therefore, that certainly had a negative influence on the ICC value. The use of two video recordings of a frontal and sagittal plane might be better for the evaluation but not practicable for clinical practice. The big advantage of the current quality rating system is the easy way to execute data collection. There is currently no “Quality First” manual containing all the information about the scoring of the different items and detailed pictures. A “Quality First” user manual could improve raters' agreement regarding the scoring system.

In the present study, the validity of the “Quality First” assessment was not assessed. Information on content validity, interpretability and internal consistency was previously reported (29). Moreover, at this stage, this study cannot suggest the “Quality First” tool for a safe return to sport. This has to be evaluated in a prospective design.

## 5 Conclusion

The missing correlation between the calculated LSI and the “Quality First” total score of the VH, SLHD, and SH tests indicates that the limb symmetry index of a hop test (jump performance symmetry) does not reflect the actual movement quality during those jump landings. It is well known that suboptimal kinematics during hop landings are a significant risk factor for repeated ACL injuries. Therefore, it is highly recommended to include the evaluation of the movement quality of hop landings in return to sport testing batteries.

The “Quality First” assessment was designed to evaluate the movement quality of a jump landing in the VH, SLHD, and SH tests in clinical practice. The results of this study suggest that the evaluation of movement quality with the “Quality First” assessment is good to excellent reliable when applied by one person. The results of the interrater reliability demonstrated fair to good reliability.

## Data Availability

The original contributions presented in the study are included in the article/Supplementary Material, further inquiries can be directed to the corresponding author.
