# Supplementary material for: Evaluation of hop test movement quality to enhance return to sport testing. A cross-sectional study
Source: Front Sports Act Living. 2024 Mar 4;6:1305817. doi: 10.3389/fspor.2024.1305817 (PMC10944867; doi:10.3389/fspor.2024.1305817)
Supplement: Supplementary file 1 [file Datasheet1.docx]

Supplementary Material

**Evaluation of Hop Test Movement Quality to Enhance Return to Sport Testing. A Cross-sectional Study**

**Melanie Weber^1^, Mirjam Müller^1^, Moritz Mathieu-Kälin^1^, Sandro Caminada^2^, Marina Häberli^2^, Heiner Baur^1*^**

^1^ Division of Physiotherapy, Department of Health Professions, Bern University of Applied Sciences, Bern, Switzerland

^2^ Altius Swiss Sportmed Center AG, Rheinfelden, Switzerland

***Correspondence:**Heiner Baur
[heiner.baur@bfh.ch](mailto:weber_melanie@gmx.ch)

Supplementary Table 1 Patient characteristics

| **Patient characteristics** |  |  |  |  |  |  |
| --- | --- | --- | --- | --- | --- | --- |
|  | **N** | **Mean** | **Median** | **SD** | **Min** | **Max** |
| Sex |  |  |  |  |  |  |
| female | 16 |  |  |  |  |  |
| male | 18 |  |  |  |  |  |
|  |  |  |  |  |  |  |
| Age (years) |  | 24.2 | 22.5 | 8.2 | 13 | 43 |
| Height (cm) |  | 173.4 | 174.5 | 8.5 | 158 | 190 |
| Weight (kg) |  | 71.2 | 69.0 | 12.1 | 54 | 106 |
| Time after OP (month) |  | 9.4 | 9.0 | 2.9 | 5 | 17 |
| Tegner Score (max: 10) |  | 7.6 | 7.00 | 1.1 | 6 | 10 |
| Graft type: |  |  |  |  |  |  |
| SemiT | 29 |  |  |  |  |  |
| Quadriceps | 5 |  |  |  |  |  |
|  |  |  |  |  |  |  |
| Additional injuries: | 21 |  |  |  |  |  |
| lateral meniscus tear | 10 |  |  |  |  |  |
| medial meniscus tear | 11 |  |  |  |  |  |
| LCL | 1 |  |  |  |  |  |
| MCL | 3 |  |  |  |  |  |
| cartilage damage | 2 |  |  |  |  |  |
| bone bruise | 1 |  |  |  |  |  |
| other | 3 |  |  |  |  |  |

N: Number, SD: Standard deviation, Min: Minimum, Max: Maximum, SemiT: M. Semitendinosus, LCL: Lateral collateral ligament, MCL: Medial collateral Ligament

Supplementary Figure 1. "Quality First" assessments (Vertical Hop, Single Leg Hop for Distance, Side Hop)

Supplementary Table 2 Descriptive statistics overall "Quality First" total scores

| **"Quality First" overall total scores** | | | | | | | |
| --- | --- | --- | --- | --- | --- | --- | --- |
|  |  |  |  |  |  | **95 % CI** | |
|  |  | **N** | **Mean** | **SD** | **SE** | **Lower Bound** | **Upper Bound** |
| VH | Interrater | 150 | 16.1 | 2.8 | 0.2 | 15.64 | 16.55 |
|  | intrarater | 100 | 19.8 | 2.6 | 0.3 | 19.25 | 20.25 |
|  |  |  |  |  |  |  |  |
|  |  |  |  |  |  |  |  |
| SLHD | Interrater | 150 | 15.3 | 2.6 | 0.2 | 14.91 | 15.73 |
|  | intrarater | 100 | 20.1 | 2.1 | 0.2 | 19.67 | 20.49 |
|  |  |  |  |  |  |  |  |
|  |  |  |  |  |  |  |  |
| SH | Interrater | 150 | 10.6 | 2.6 | 0.2 | 10.19 | 11.03 |
|  | intrarater | 100 | 14.6 | 1.8 | 0.2 | 14.25 | 14.97 |
|  |  |  |  |  |  |  |  |

VH: Vertical hop, SLHD: Single-leg hop for distance, SH: Side hop, N: Number, SD: Standard deviation, SE: Standard error, CI: Confidence interval

Supplementary Table 3 Descriptive Statistics LSI and "Quality First"

| **Descriptive statistic LSI and "Quality First"** | | | | | | | | | | | |
| --- | --- | --- | --- | --- | --- | --- | --- | --- | --- | --- | --- |
|  |  | **LSI** | | | | | **“Quality First” total score** | | | | |
|  |  |  |  |  | **95 % CI** | |  |  |  | **95 % CI** | |
|  | **N** | **Mean** | **SD** | **SE** | **Lower Bound** | **Upper Bound** | **Mean** | **SD** | **SE** | **Lower Bound** | **Upper Bound** |
| VH, LSI ≥ 90 % | 12 | 98.8 | 5.9 | 1.7 | 95.42 | 102.09 | 19.3 | 3.2 | 3.2 | 17.06 | 21.16 |
| VH, LSI < 90 % | 13 | 76.1 | 10.7 | 3.0 | 70.29 | 81.97 | 20.0 | 2.1 | 0.6 | 18.85 | 21.15 |
|  |  |  |  |  |  |  |  |  |  |  |  |
| SLHD, LSI ≥ 90 % | 21 | 99.6 | 5.0 | 1.1 | 97.44 | 101.69 | 20.0 | 2.4 | 0.5 | 18.96 | 21.03 |
| SLHD, LSI < 90 % | 4 | 85.8 | 5.2 | 2.6 | 80.73 | 90.94 | 20.8 | 1.7 | 0.9 | 19.08 | 22.423 |
|  |  |  |  |  |  |  |  |  |  |  |  |
| SH, LSI ≥ 90 % | 13 | 102.3 | 9.6 | 2.7 | 97.02 | 107.02 | 14.5 | 1.6 | 0.4 | 13.61 | 15.309 |
| SH, LSI < 90 % | 12 | 76.8 | 13.1 | 3.8 | 69.38 | 84.20 | 14.6 | 1.7 | 0.5 | 13.63 | 15.53 |

V: Vertical hop, SLHD; Single-leg hop for distance, SH: Side hop, LSI: Limb symmetry index, N: Number, SD: Standard deviation, SE: Standard error, CI: Confidence interval

Supplementary Table 4 T-test independent sample

| **T-Test independent sample (total score "Quality First" and LSI ≥ 90 resp. <90 %)** | | | | |
| --- | --- | --- | --- | --- |
|  |  |  | **95 % CI** | |
|  | **t** | **p-value** | **Lower Bound** | **Upper Bound** |
| Welch Test (unequal variances): |  |  |  |  |
| Vertical Hop | 0.60 | 0.55 | -1.64 | 2.98 |
| Single leg Hop for distance | 0.75 | 0.49 | -1.75 | 3.25 |
| T-test (equal variances): |  |  |  |  |
| Side hop | 0.19 | 0.85 | -1.22 | 1.46 |

CI: Confidence interval

**

**Supplementary Figure 2.** Correlation “Quality First” total scores and LSI (LSI: Limb symmetry index, VH: vertical Hop, SLHD: Single-leg hop for distance, SH: Side hop)

**

Supplementary Figure 3. Dataset first analysis Side Hop, Rater 1

Supplementary Figure 4. Dataset first analysis Single Leg Hop for Distance, Rater 1

Supplementary Figure 5. Dataset first analysis Vertical Hop, Rater 1

Supplementary Figure 6. Dataset second analysis Side Hop, Rater 1

Supplementary Figure 7. Dataset second analysis Single Leg Hop for Distance, Rater 1

Supplementary Figure 8. Dataset second analysis Vertical Hop, Rater 1

Supplementary Figure 9. Dataset Side Hop, Rater 2

Supplementary Figure 10. Dataset Single Leg Hop for Distance, Rater 2

Supplementary Figure 11. Dataset Vertical Hop, Rater 2

Supplementary Figure 12. Dataset Side Hop, Rater 3

Supplementary Figure 13. Dataset Single Leg Hop for Distance, Rater 3

Supplementary Figure 14. Dataset Vertical Hop, Rater 3

Supplementary Figure 15. Dataset Side Hop, Rater 1 after consensus

Supplementary Figure 16. Dataset Single Leg Hop for Distance, Rater 1 after consensus

Supplementary Figure 17. Dataset Vertical Hop, Rater 1 after consensus

Supplementary Figure 18. Dataset correlation analysis Side Hop

Supplementary Figure 19. Dataset correlation analysis Single Leg Hop for Distance

Supplementary Figure 20. Dataset correlation analysis Vertical Hop
